# Granzyme B and melittin in cancer immunotherapy: molecular mechanisms and therapeutic perspectives in head and neck cancers

**DOI:** 10.3389/fimmu.2025.1628014

**Published:** 2025-07-22

**Authors:** Adam Majchrzak, Filip Lewandowski, Rafał Hrynkiewicz, Agata Poniewierska-Baran, Dominika Bębnowska, Paulina Niedźwiedzka-Rystwej

**Affiliations:** ^1^ Department of Infectious, Tropical Diseases and Immune Deficiency, Pomeranian Medical University in Szczecin, Szczecin, Poland; ^2^ Center for Experimental Immunology and Immunobiology of Infectious and Cancer Diseases, University of Szczecin, Szczecin, Poland; ^3^ Institute of Biology, University of Szczecin, Szczecin, Poland; ^4^ Regional Centre for Digital Medicine, Pomeranian Medical University in Szczecin, Szczecin, Poland

**Keywords:** granzyme B, melittin, hNSC, cytotoxicity, NK cells, CTL

## Abstract

Granzyme B (GZMB) and melittin are potent cytotoxic agents with promising applications in cancer immunotherapy, particularly in head and neck squamous cell carcinoma (HNSC). GZMB, secreted by cytotoxic T lymphocytes and natural killer (NK) cells, induces apoptosis through caspase activation and mitochondrial disruption. Its expression in HNSC correlates with both improved prognosis and, paradoxically, immune suppression via regulatory T cells. Melittin, a peptide derived from bee venom, exerts anticancer effects by disrupting cancer cell membranes, inducing oxidative stress, and activating apoptotic pathways. While effective, its non-specific cytotoxicity poses a therapeutic challenge, which is being addressed through targeted delivery systems, such as nanoparticles and liposomes. This review highlights the distinct yet potentially complementary roles of GZMB and melittin in modulating tumor cell death and the tumor microenvironment. We also discuss mechanisms of resistance, including expression of granzyme inhibitors (e.g., PI-9), altered membrane dynamics, and G2/M cell cycle arrest. Combining the specificity of immune-mediated GZMB action with the broad cytotoxicity of melittin may offer synergistic benefits in future therapies. Understanding these molecules’ mechanisms provides a foundation for novel immunotherapeutic strategies in the treatment of HNSC and other solid tumor.

## 1 Introduction

Granzyme B (GZMB), also known as granzyme β, is a caspase-like serine proteolytic enzyme, released from granules of natural killer cells (NK cells) and cytotoxic T cells to kill virus-infected and tumor cells. GZMB has a tryptase activity, which means that it cleaves the protein chain between arginine and lysine. GZMB molecular mass is 27–32 kDa and has been found in humans, rats and mice (1–3). It can be expressed in hematopoietic origin cells, i.e. lymphocytes, neutrophils, basophils, dendritic cells, mast cells or activated macrophages (4–6), but also in non-haematopoietic origin cells, like keratinocytes (7), primary spermatocytes (8), synovium (9), chondrocytes (10), hepatocyte (11), type II pneumocytes (12) and others (13).

Granzymes, including granzyme B, are safely contained within the cytotoxic granules of CTL and NK cells so as not to damage the host cell. Upon recognition of the target by CTL or NK cells, cytotoxic granules (containing both granzyme and perforin, a key granzyme transporter) migrate along microtubules to the plasma membrane, near the target, where they are then secreted into the “immunological synapse” to promote rapid apoptotic cell death of the infected or cancerous cell (14). As recent discoveries have shown, tumor growth and immune evasion during cancer development may depend on the activity and expression of granzyme B (GZMB) in immune cells (13). The importance of the perforin/granzyme pathway in cancer prevention, as well as in immune homeo-stasis, has been clearly demonstrated in perforin mice deficient studies. The study by Metkar et al. (15) in granzyme A and granzyme B mice deficient, has revealed a possible new mode of action of these important proteases, as effectors of inflammation.

In recent years, growing interest has also been directed toward melittin, a bioactive peptide derived from bee venom, which demonstrates both direct cytotoxic and immunomodulatory properties. Melittin is a 26-amino-acid amphipathic molecule capable of selectively disrupting cancer cell membranes due to their altered lipid composition (16, 17). Upon membrane insertion, melittin forms pores and causes destabilization of lipid bilayers, leading to loss of cellular integrity, ion imbalance, and in many cases, apoptosis (18–20). In addition to its lytic activity, melittin modulates intracellular signaling pathways—inducing mitochondrial dysfunction, cytochrome c release, caspase activation (21–23), and inhibiting NF-κB-mediated survival signaling (24, 25). These actions position melittin not only as a potent cytotoxin but also as an apoptosis inducer and immunostimulatory agent.

Melittin has also been shown to modulate the tumor microenvironment (TME) by inhibiting angiogenesis and enhancing immune cell infiltration (23, 26). Although its therapeutic potential is promising, its non-specific cytotoxicity poses a challenge, prompting the development of delivery systems such as liposomes, nanoparticles, or tumor-targeting fusion proteins (27–29). Importantly, melittin may synergize with immune effector mechanisms—such as granzyme B-dependent cytotoxicity—making it an attractive candidate for combination immunotherapy (30).

This review further aims to explore and compare the distinct, yet potentially complementary roles of granzyme B and melittin in tumor cell elimination and their translational potential in cancer immunotherapy.

## 2 Biology and mechanism of granzyme B

### 2.1 Detailed structure

**Figure 1:**
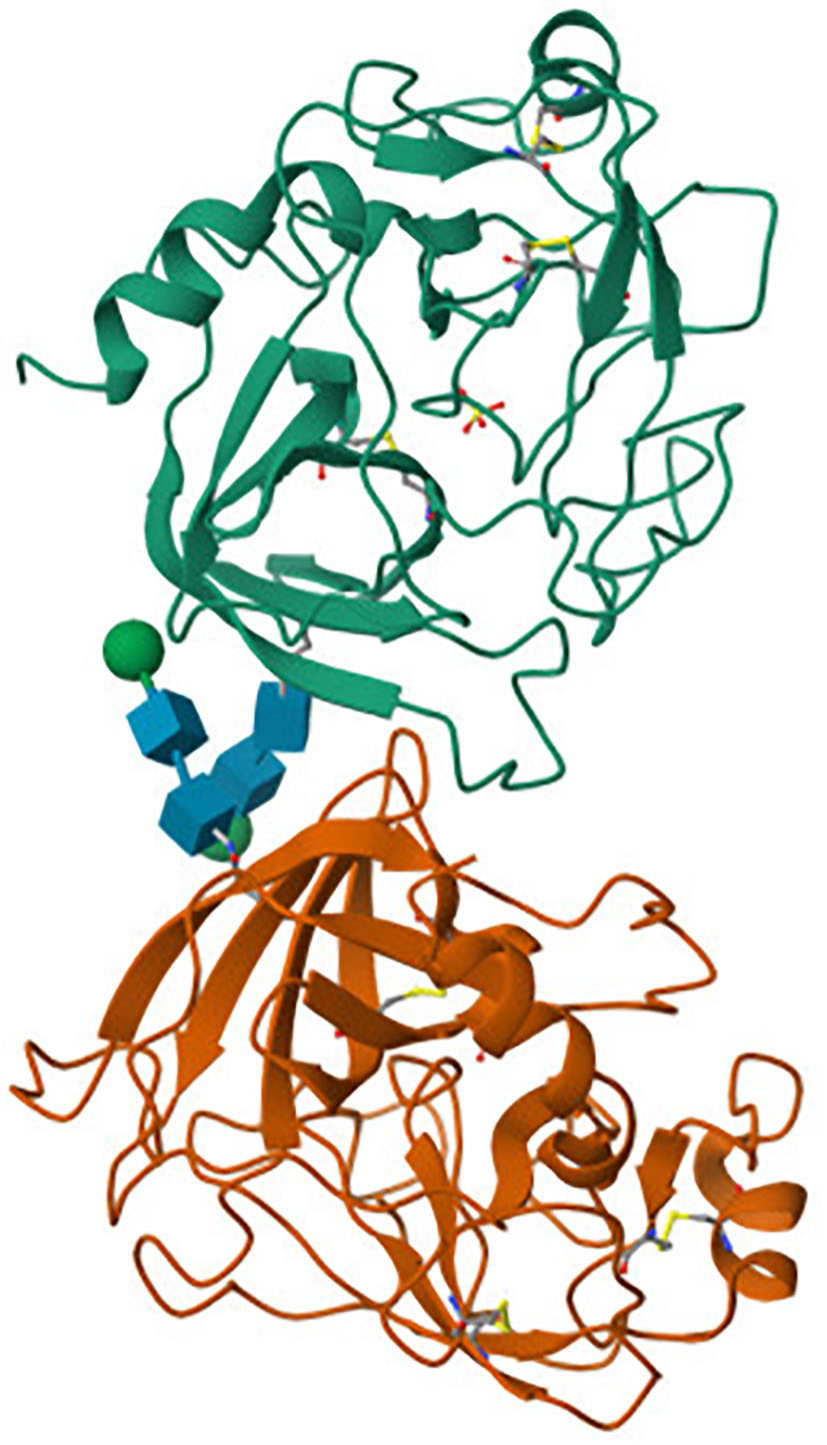
Crystal structure of human granzyme B obtained by X-ray diffraction (31, 32). The tertiary structure of human granzyme B consists of two globular β-barrel domains (green and orange), connected by linker segments (blue). The overall fold is characteristic of the chymotrypsin-like serine protease family. While the active site residues and substrate-binding pockets are not annotated here, their spatial arrangement within the β-barrels enables granzyme B to efficiently recognize and cleave intracellular substrates during immune-mediated cytotoxicity.

Granzyme B (**Figure 1**) is a serine protease with a single chain and single domain, identified and purified from granules in 1991 by Poe et al. (33) The crystal structure of human GZMB is specific - two six-stranded ß-barrels are connected by three trans-domain segments (34, 35). The secondary structure is based on a helical loop between Ala56 and Cys58, a helix spanning residues Asp 165 to Leu172, and a long C-terminal helix from Phe234 to Arg244. The peptide bond between Pro224 and Pro225 is in a cis conformation, which orients the positively charged side chain of Arg 226 at the S1 position (35). Granzyme B, like other known human granzymes (A, H, K, and M) along with myeloid serine proteases (such as cathepsin G), is a member of the chymotrypsin superfamily (36). GZMB is synthesized as a pre-pro-enzyme. For the first time, the human pre-pro-GZMB mRNA was identified in leukocytes in 1987 by Schmid and Weissmann (37) and human GZMB cDNA was cloned just a year later by two independent teams, Krahenbuhl et al. (38) and Trapani et al. (39). Pre-pro-GZMB signal peptide (i.e., 18-amino-acid long pre-N-terminal portion), which directs the nascent polypeptide chain of the protein to the endoplasmic reticulum (ER), is cotranslationally removed. The resulting pro- GZMB is transported by ER-derived vesicles to the Golgi apparatus (GA) and then directed to secretory granules, where they become activated (39, 40), by i.e. co-segregated dipeptidyl peptidase I (DPPI; cathepsin C) (39, 41, 42), lysosomal cathepsin H (43), or by a proteolytic activation mechanism (44, 45). The human GZMB gene (GZMB) is located on chromosome 14q11.2 (46), together with the granzyme H (GZMH), cathepsin G (CTSG) and mast cell chymase (CMA1) genes. The GZMB gene shows polymorphism in various racial groups (47).

### 2.2 Mechanism of action and inducing apoptosis

It is known that GZMB induces apoptotic cell death through NK and CTL. Granzymes are stored in NK and CTL cells secretory granules, along with the chondroitin sulfate-containing proteoglycan serglycin (SG) and are co-secreted with SG in a in two types of macromolecular complex (31, 48). First, with small molecular sizes (approximately 4–8 GZMB molecules), or bigger with 32 GZMB molecules (31). The activity of the free GZMB molecule differs from SG-bound GZMB. When GZMB binds to SG, its surface charge is substantially neutralized, whereas the free GZMB subunit can interact with various negatively charged groups exposed on the cell surface, like phospholipids and glycosaminoglycans (49). Once in the cytosol, Granzyme B degrades substrate proteins by proteolysis and leads to programmed cell death.

In a classical GZMB activation, the antigen-presenting cells - APC, due to the major histocompatibility complex (MHC), which triggers calcium influx through the phospho-lipase C (PLC) pathway, make the pathogenic antigen visible to CTL cells (13). It is mainly through increased calcium levels that cytotoxic granules are mobilized to polarize towards the immunological synapse, where granzymes and perforin are released in the process of degranulation. This signal forces CTL cells to migrate towards the antigen, approach it and create an immunological synapse, which begins the process of killing the target cell. The situation is different for NK cells, which do not require antigen recognition with the participation of MHC, which is of great importance for the efficient elimination of virally infected cells, as well as tumor cells (50). A contact zone is created between the target cell and the killer cell, and the so-called adhesion ring is created, which causes polarization and redirection of the granules with GZMB towards this location (38). Cytotoxic molecules such as GZMB and perforins can also be activated by pro-inflammatory cytokines, like type I interferon (IFN) (51), IL-18 (52), or IL-2 (53) and IL-15 (54) via the JAK1/3-STAT3/5 pathway. GZMB transcription can be regulated by activating transcription factor (ATF), cyclic AMP–responsive element-binding protein (CREB) interaction, activator protein-1 (AP-1), Ikaros, core-binding factor (CBF/PEBP2), Runx3 and T-box factors (T-bet) (55–57). Interestingly, *in vitro* studies by Karreci et al. (58) have shown that activated human Tregs generate GZMB along with its inhibitor, serine protease inhibitor 9 (Serpin9), which is a type of cell defense mechanism against self-destruction. It should be noted, however, that high levels of Serpin9 were not able to completely protect the Tregs cell population from undergoing apoptosis.

**Figure 2:**
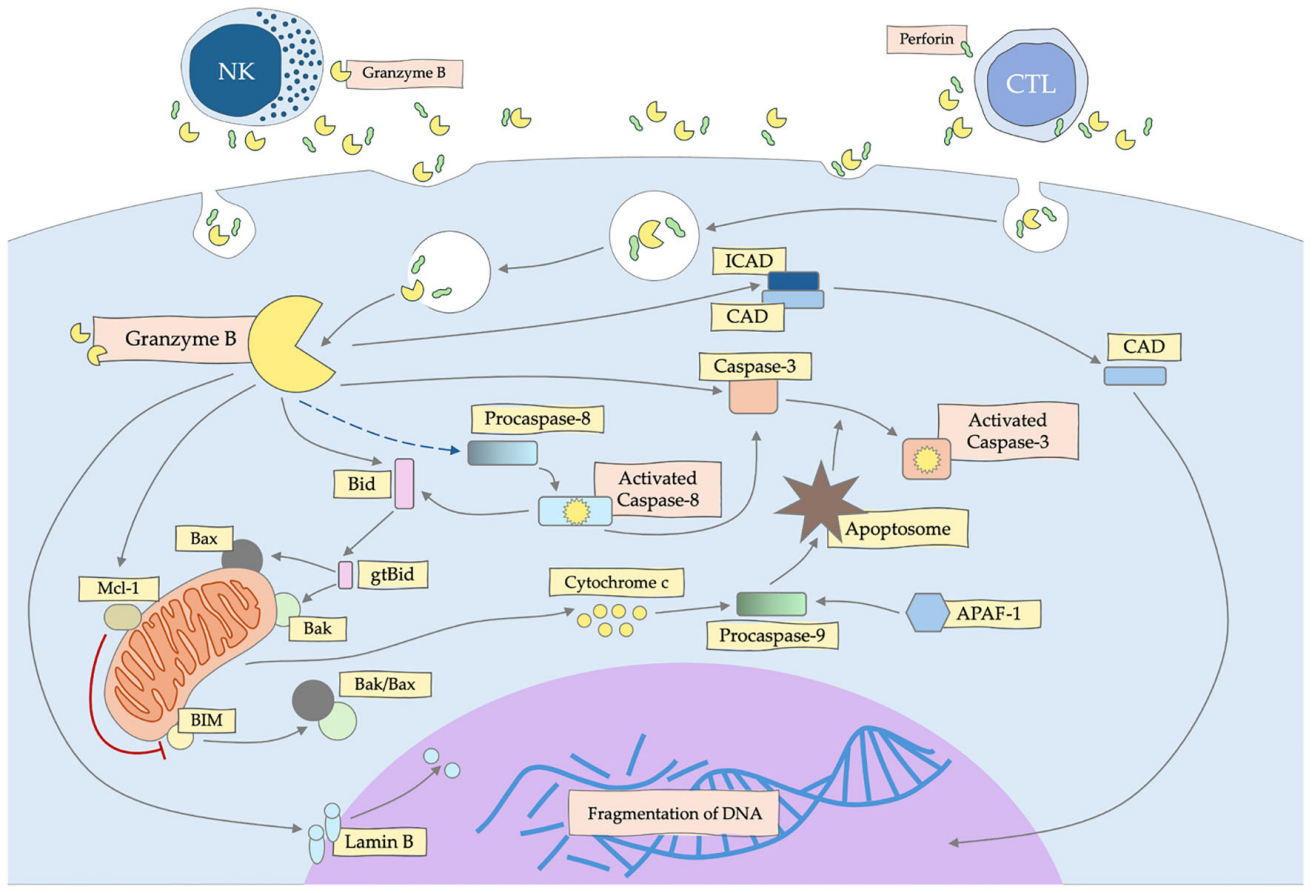
Mechanism of apoptosis induction by granzyme B (GZMB) secreted by NK and CTL cells. GZMB activates the caspase and mitochondrial pathway, leading to DNA fragmentation and death of the target cell.

Inducing apoptotic cell death by granzyme B (**Figure 2**) is crucial to eliminating threats, such as virus-infected or cancer cells (4). GZMB is capable of activation the procaspase-3 and -7, but also procaspase-2, -6 -8, -9 and -10 (59–61). Some indicate that GZMB triggers apoptosis through changes of the outer mitochondrial membrane (OMM) and not only caspases (62). Granzyme B can cleave multiple extracellular matrix components, including fibronectin, vitronectin, as well as cytoskeleton elements, such as tubulin or filamin (63, 64). This, in turn, leads to damage to the nuclear lamina by GZMB, thanks to which other granzymes have an easy nuclear route by the nuclear pore complexes, triggering many critical intranuclear molecular processes (65). It also has been demonstrated that GZMB can bind to the target cell surface and enter the cell via endocytosis (66). What is interesting about the concept of GZMB involvement in the induction of apoptosis, is that T lymphocyte (CTL)-dependent cytotoxicity can be effective even in the absence of GzmA or GZMB, which has raised the question of the role of granzymes in cell death and other processes of the immune response (67).

### 2.3 Role in immune response

It is well known that GZMB has antipathogenic properties, depending on the induction of the apoptosis process, but interestingly there are studies indicating that GZMB may also act antipathogenic in a way independent of cell apoptosis. GZMB cleaves multiple viral proteins essential for viral replication and/or evading host cell targeting, including herpes simplex virus (HSV) and varicella-zoster virus (VZV) ORF4 and ORF62, which can inhibit both intrinsic and extrinsic apoptosis pathways (68, 69), making this action of GZMB a secondary mechanism for inhibiting viral spread. Moreover, GZMB can block viral replication in a caspase-dependent (70) as well as caspase-independent manner (71).

GZMB can affect bacterial and parasitic cells by activating the production of reactive oxygen species (ROS), the anions of which disrupt the mitochondrial potential, inducing parasite apoptosis, or in the case of bacteria, cause irreversible damage through super-oxide dismutase (SOD) and inhibit the response to oxidative stress in aerobic bacteria (72).

Many studies describe the activity of GZMB not only in the apoptosis, but also in the inflammation process, pathogenesis of autoimmune diseases and even in development of cancer (73). Indeed, the major granzyme players (GZMB and GzmA) have been shown to regulate angiogenesis and both vascular or extracellular matrix remodeling, which are important not only in tissue repair or organ development, but also in cancer progression (13). Until recently, it was believed that GZMB activity protects us from cancer or reduces tumor activity, but data are emerging that may challenge this. GZMB has been localized in many cancer cells, i.e. breast (74), lung (75) and urothelial carcinomas (76), nasal-type NK/T-cell lymphoma (77), oral squamous cell carcinoma (78). Pan-cancer analysis also showed higher GZMB mRNA expression in many cancers (compared to normal tissues), such as cholangiocarcinoma, glioblastoma multiforme, kidney renal clear cell carcinoma, gastric adenocarcinoma, as well as head and neck squamous cell carcinoma (13).

Basically, the role of granzyme B depends on the location of its action, we distinguish three locations: intracellular, cell surface and extracellular (79). In extracellular matrix (ECM) modulation, GZMB impacting cellular adhesion and migration, by disrupting cellular-vitronectin interactions, which is important for the migration of primed CD8+ CTLs (64). As was shown by Lindner et al. (79), B cells secrete regulatory molecules, such as IL-10, IL-12, but also GZMB, which weaken the response of T cells by damaging the ζ chain in their TCR receptor. The efficiency of GZMB -dependent apoptosis of cancer cells depends mainly on the amount of GZMB that is delivered to their cytoplasm.

## 3 Biology and mechanism of melittin

### 3.1 Detailed structure

**Figure 3:**
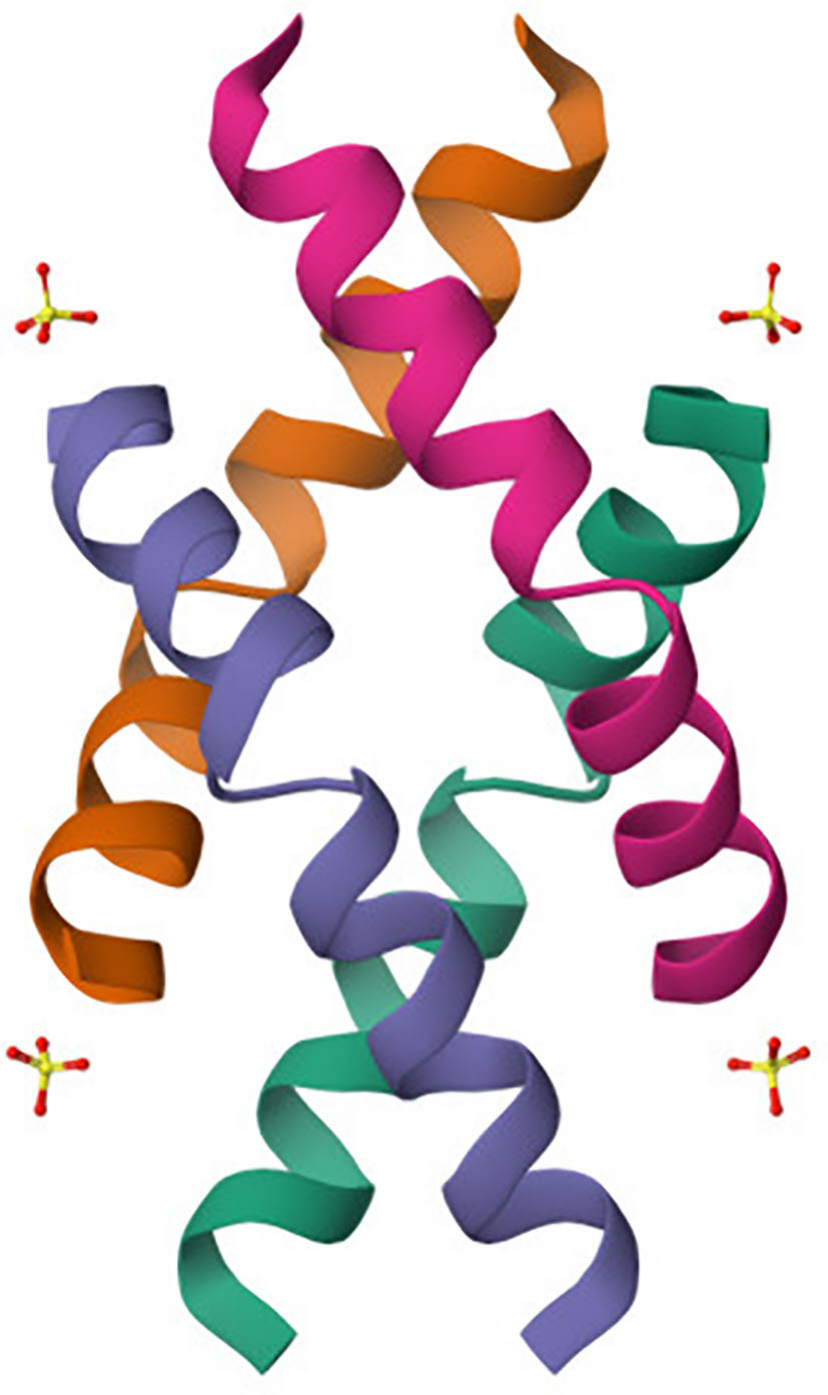
Crystal structure of melittin obtained by X-ray diffraction (80). The crystal structure of melittin reveals a helical arrangement typical of its amphipathic nature. The peptide forms a bundle of α-helices (colored individually) characteristic of its membrane-active conformation. This conformation facilitates insertion into lipid bilayers via the “barrel-stave” model, contributing to its cytolytic function. The charged and hydrophobic faces of the helices enable selective interaction with negatively charged cancer cell membranes, underpinning melittin’s pore-forming and pro-apoptotic activity.

Melittin (**Figure 3**), a principal component of bee venom, is a small linear amphipathic peptide made up of 26 amino acids (17). Its structure plays a crucial role in its biological function and interaction with cellular membranes (18). The peptide has a dual character, where the N-terminal region is predominantly hydrophobic and capable of interacting with lipid bilayers, while the C-terminal region is positively charged, contributing to its water solubility and ionic interactions with the membrane surfaces. In an aqueous environment, melittin tends to aggregate and can form tetramers (19). However, when it interacts with lipid membranes, it undergoes significant conformational changes, often transitioning into a helical structure that inserts itself into the lipid bilayer. This insertion is driven by the amphipathic nature of melittin, allowing it to align hydrophobic residues with the membrane interior while keeping hydrophilic residues exposed to the aqueous phase. The peptide’s membrane-disrupting activity arises from its ability to form pores, a mechanism known as the “barrel-stave” model (20). In this arrangement, multiple melittin molecules align in parallel to create a transmembrane channel, disturbing membrane integrity and causing cellular lysis. The effectiveness of pore formation depends on peptide concentration and the lipid composition of the target membrane (26). Melittin’s structural features not only contribute to its membrane-lytic activity but also allow it to interact with various cellular proteins and induce signaling pathways, including the activation of phospholipase A2 and modulation of ion channels (30). These interactions underlie its broad-spectrum antibacterial and anticancer activities, as melittin has been shown to inhibit cell proliferation and induce apoptosis in numerous cancer cell lines (30, 81, 82).

### 3.2 Mechanism of action and inducing apoptosis

Melittin operates through a multifaceted mechanism that targets cellular membranes, disrupts signal transduction, and initiates cell death pathways. At the core of melittin’s cytotoxic activity is its ability to penetrate and disturb the integrity of lipid bilayers (83). The peptide, due to its amphipathic structure, binds preferentially to negatively charged phospholipids present in microbial and cancer cell membranes. This selective affinity leads to the formation of pores and destabilization of the lipid bilayer. Upon binding to the membrane surface, melittin initially forms an α-helical structure that facilitates its insertion into the hydrophobic core of the bilayer. This membrane insertion can occur through several models, including the “carpet model” and the “barrel-stave model” (17). In the carpet model, melittin peptides aggregate on the membrane surface, covering it like a carpet, until a critical concentration is reached, causing the membrane to disintegrate (84). In contrast, the barrel-stave model describes the peptide inserting perpendicularly into the membrane to form transmembrane pores, leading to ion leakage, loss of membrane potential, and ultimately cell lysis (21). Beyond membrane disruption, melittin’s mechanism extends to the activation of intracellular pathways that drive apoptosis. A key aspect of this apoptotic action is mitochondrial dysfunction (22). Melittin can induce mitochondrial membrane depolarization, which results in the release of cytochrome c into the cytosol. This release is a crucial step in the activation of the intrinsic apoptotic pathway, triggering the caspase cascade (23). Caspase-9, an initiator caspase, is activated first, subsequently activating effector caspases such as caspase-3 and caspase-7. These caspases execute the apoptotic program by cleaving essential cellular substrates, leading to DNA fragmentation and cell morphological changes, including shrinkage and membrane blebbing (24, 25). Melittin disrupts key signaling pathways involved in cell survival. The NF-κB pathway, a regulator of anti-apoptotic genes, is inhibited by melittin. By preventing the nuclear translocation of NF-κB, melittin reduces the transcription of genes that promote cell survival and proliferation (25). This inhibition not only facilitates apoptotic cell death but also sensitizes cancer cells to other therapeutic agents (17). Additionally, melittin can induce oxidative stress by enhancing the generation of reactive oxygen species (ROS), which causes further damage to cellular com-ponents and amplifies the apoptotic signal (85). Another notable effect of melittin is its ability to activate enzymes such as phospholipase A2 (PLA2) (30). The activation of PLA2 leads to the release of arachidonic acid and the production of pro-inflammatory and pro-apoptotic lipid mediators. These mediators contribute to cell death and inflammation, which can be beneficial in targeting cancer cells but pose a risk of toxicity to normal tissues if not adequately controlled (86, 87).

## 4 Comparison of melittin and granzyme B mechanisms

Melittin is primarily structured to disrupt lipid bilayers, making it a general cytotoxic agent that affects a wide range of cells, including bacteria and mammalian cells (88). On the other hand, Granzyme B is an enzymatic molecule, designed specifically to cleave proteins within target cells, triggering apoptosis (89). The properties of the two substances are compared in **Table 1**.

**Table 1:** Comparison of Melittin and Granzyme B.

Characteristic	Melittin	Granzyme B
Primary Structure	Amphipathic peptide designed to disrupt lipid bilayers.	Enzymatic molecule targeting intracellular proteins.
Mechanism of Action	Disrupts cellular membranes, forms pores, and causes loss of membrane integrity.	Cleaves specific intracellular proteins (e.g., caspase-3, BID) to trigger apoptosis.
Apoptotic Induction	Indirect: Mitochondrial membrane depolarization and ROS generation; excessive concentrations may cause necrosis.	Direct: Cleaves pro-apoptotic proteins, activating caspases and the intrinsic apoptotic pathway.
Specificity	Non-selective: Affects any cell with lipid bilayers.	High specificity: Targets virus-infected or tumor cells via immune cell delivery.
Effects on Immune Response	Activates phospholipase A2, releasing pro-inflammatory mediators, often exacerbating inflammation.	Central to immune defense; works synergistically with perforin to eliminate harmful cells.
Therapeutic Challenges	High non-selective cytotoxicity limits clinical use; delivery systems (e.g., liposomes, nanoparticles) are being explored.	Stability and delivery challenges; potential for targeted immune-based therapies.
Therapeutic Applications	Broad-spectrum antimicrobial and anticancer agent; risk of damage to healthy tissues.	Immune surveillance, cancer therapy, and treatment of infectious diseases.
Evolutionary Role	Broad-spectrum membrane disruptor.	Highly specific immune effector molecule.
Key Risks	Risk of necrosis at high concentrations; inflammatory effects.	Limited unintended tissue damage; minimal risk of chronic inflammation.

Melittin’s primary mode of action is through the disruption of cellular membranes. Its amphipathic structure allows it to insert into lipid bilayers, forming pores and causing a loss of membrane integrity (83). This action leads to an uncontrolled influx and efflux of ions, resulting in osmotic imbalance, cell lysis, or in some cases, the initiation of apoptotic pathways due to mitochondrial damage and oxidative stress (88, 90). In contrast, Granzyme B functions intracellularly. After being delivered into the target cell through perforin-created pores, Granzyme B cleaves key substrates such as caspase-3 and BID (79). This cleavage directly activates the intrinsic apoptotic pathway, leading to controlled cell death without widespread membrane damage. Major distinction between the two molecules lies in their specificity (82). Melittin’s action is non-selective, meaning it can affect any cell type that comes into contact with its disruptive capabilities (91). This property makes melittin highly effective as an antimicrobial and anticancer agent but also poses a significant risk of damaging healthy cells, which limits its therapeutic use (88). Granzyme B, however, exhibits high specificity. It targets cells recognized as harmful, such as virus-infected or tumor cells, and is delivered with precision by immune effector cells. This specificity allows Granzyme B to play a crucial role in immune surveillance while minimizing damage to surrounding healthy tissues (22). While both molecules can induce apoptosis, they do so in distinct ways (27, 91).

Melittin indirectly triggers apoptosis by causing mitochondrial membrane depolarization and the generation of reactive oxygen species (ROS) (21). These disturbances can lead to the release of pro-apoptotic factors such as cytochrome c, activating the intrinsic apoptotic pathway (92). However, if melittin’s concentration is too high, cells may die through necrosis due to overwhelming membrane disruption (93). Granzyme B induces apoptosis more directly and efficiently. By cleaving specific pro-apoptotic proteins, it activates caspases and orchestrates a rapid and organized cell death process. This direct pathway minimizes the risk of inflammation or unintended tissue damage (22).

Melittin also influences immune responses but does so in a broad and often inflammatory manner. It can activate phospholipase A2 and release pro-inflammatory mediators, which may exacerbate inflammatory conditions if not carefully controlled (28, 88). Granzyme B, conversely, is a central player in immune defense. It works synergistically with other components of the immune system, such as perforin, to target and eliminate pathogenic or malignant cells in a regulated manner. This function highlights its role in maintaining immune homeostasis and preventing chronic inflammation (73, 94).

The therapeutic use of melittin is limited by its non-selective cytotoxicity, which poses a significant challenge for clinical application. Researchers are exploring delivery systems such as liposomes or nanoparticles to target cancer cells specifically and minimize harm to normal tissues (29, 95). Granzyme B, with its targeted apoptotic mechanism, holds promise for immune-based therapies. Strategies that harness or enhance the activity of Granzyme B are being developed for treating cancers and infectious diseases, although delivery and stability remain areas of active research (96).

Melittin and granzyme B are effective cytotoxic agents, their mechanisms reflect different evolutionary purposes. Melittin is a broad-spectrum membrane disruptor, whereas Granzyme B is a highly specific immune effector molecule (**Table 1**). These differences have profound implications for their potential therapeutic applications and the strategies needed to optimize their use.

## 5 Role in oncology

### 5.1 Granzyme B

#### 5.1.1 Expression and clinical relevance of GZMB in head and neck cancers

Granzyme B (GZMB) is a key cytotoxic element of the immune system, playing a fundamental role in killing cancer cells by inducing apoptosis (13). However, its function in head and neck cancers, particularly in head and neck squamous cell carcinoma (HNSC), is more complex than first thought (78, 97, 98). Analyses of available data, covering a broad spectrum of tumor, show that GZMB expression in head and neck squamous cell carcinoma (HNSC) is increased, compared to normal tissues. This is consistent with the classical model in which GZMB acts in cytotoxic T lymphocytes (CTL) and NK cells, leading to the elimination of tumor cells. However, the GZMB expression differ between different cases of the same cancer type. In HNSC, for example, it has been reported that GZMB expression levels can vary significantly between patients, suggesting the influence of a various microenvironmental or genetic factors (99–101).

One important finding is that in HNSCs, higher GZMB expression may be associated with the presence of regulatory T cells (Treg), which also express FoxP3. Treg are usually associated with immunosuppression and their presence in tumor may limit the effectiveness of the immune response (13, 102). In studies of HNSCs, it has been shown that patients with elevated GZMB and FoxP3 expression have a higher probability of survival (103–105). This may imply that GZMB in Treg plays a more complex role, not only in the elimination of tumor cells, but also in the modulation of the immune response.

*Glioblastoma multiforme* (GBM) also showed elevated GZMB expression. The results indicate that GBM has high GZMB expression, which may be related to an intense immune response at the level of the tumor microenvironment (106, 107). However, as in the case of HNSCs, this expression may have an ambivalent role - on the one hand to promote elimination of tumor cells, and on the other hand to promote tumor resistance mechanisms, especially in the context of Treg-associated immunosuppression.

Another interesting aspect is the correlation between GZMB expression and tumor mutational burden (TMB) index in head and neck cancers. High GZMB expression in HNSCs was positively correlated with higher TMB values, suggesting that such tumor may be more amenable to immunotherapy. High TMB is a marker indicating an in-creased number of mutations, which may make the tumor more immunogenic and thus more susceptible to therapies based on the immune response. In the context of GZMB, HNSC tumor with high levels of GZMB and TMB may be more sensitive to immunotherapies that stimulate the activity of cytotoxic T cells and NK cells (108–110).

GZMB expression in HNSCs also has a direct bearing on patient prognosis. Prognostic analyses indicate that higher levels of GZMB expression correlate with better patient survival in most cases (105).

#### 5.1.2 Immunosuppressive role of granzyme B and its impact on the tumor microenvironment

The tumor microenvironment is a dynamic network of immune cells, cytokines, chemokines and extracellular matrix components that influence tumor progression (111, 112). Granzyme B plays a dual role in this environment: it can act to promote tumor control by killing tumor cells, but it can also contribute to tumor escape from the immune system through immunosuppressive effects mediated by various immune cells present in the TME (113). GZMB+ Treg are a key component of the immunosuppressive environment of TME. They can kill effector immune cells, such as CD4+ and CD8+ T cells, in a contact-dependent manner through GZMB, facilitating tumor escape from the immune system (114–116). GZMB expression in Treg is often induced by prolonged stimulation of IL-2 or TGF-β, which are found in abundance in TME (44). Some regulatory B cells subtypes produce GZMB and can inhibit T-cell proliferation by degrading key molecules in T-cell receptors, thereby reducing anti-tumor immune responses (110, 117). Plasmacytoid dendritic cells can also produce GZMB independently of perforin, leading to suppression of T-cell activation and contributing to tumor growth (116, 118). These cells can also promote the differentiation of regulatory T cells, further enhancing immunosuppression (119).

Granzyme B also affects the extracellular matrix (ECM) in TME (120). By degrading ECM proteins, GZMB releases cytokines and growth factors, such as vascular endothelial growth factor (VEGF), which promote tumor angiogenesis (121). This mechanism helps create a microenvironment that promotes tumor growth and metastasis. Cancer cells have developed mechanisms to escape GZMB-induced cell death. For example, some cancer cells increase the expression of inhibitors, such as SERPINB9, which protect them from GZMB-induced apoptosis, allowing to survive despite the presence of GZMB-secreting immune cells (120, 121).

#### 5.1.3 Granzyme B in tumor cytotoxicity and resistance mechanisms

A basic understanding of granzyme B relates to its anti-tumor function, given at least the fact that CTLs have been shown to directly kill tumor cells via the perforin/granzyme pathway (19). It has been shown that in patients with oral squamous cell carcinoma, an increased abundance of CTL cells in the tumor area was associated with a lower frequency of lymph node metastasis, a lower tumor proliferation index, and a significantly pro-longed survival time (122). On the other hand, in a study by Taghavi et al. (123), in patients with oral squamous cell carcinoma, the number of cells expressing granzyme B in the peritumoral area was associated with increased expression of this protein inside the tumor, and this correlation was more strongly expressed in patients who did not have metastases. Similar observations were also described by Pretcher et al. (124) in patients with oral cavity and lower pharynx cancer. The authors reported that in lymph nodes without metastasis, the number of CD8+ T cells and those expressing granzyme B was higher than in lymph node tissues with metastasis. Recently, Ito et al. (125) evaluated the significance of different subpopulations of tumor infiltrating lymphocytes (TILs), including granzyme B-positive ones, in patients with oral squamous cell carcinoma. As a result, it was shown that in a group of patients with many granzyme B-exposing TILs in the IM (invasive margin) area, DFS and OS rates were higher than in those with low numbers of these cells. These reports suggest that granzyme B contributes to a better prognosis in patients with these tumors. However, there have also been conflicting reports. Aggarwal et al. (126) found that patients with oral squamous cell carcinoma had an increased frequency of Treg cells expressing granzyme B compared with healthy controls. It has also been reported that a higher survival rate in patients with uveal melanoma occurred with low levels of granzyme B transcription compared with high levels, which strongly correlated with the expression of the Treg-associated transcription factor FoxP3 in some patients (13).

However, the use of granzymes as potential therapeutics can be problematic due to different strategies for escaping the immune system response are observed in cancer cells. Mechanisms for avoiding cytotoxicity can vary. Blocking pore formation for granzyme B may occur because of increased ordering of plasma membrane lipids resulting in reduced perforin binding, externalization of phosphatidylserine may result in its aggregation, or reduced cell stiffness may impede efficient perforin pore formation (112). On the other hand, the resistance of tumor cells to the cytotoxic effect of lymphocytes may result from the degradation of perforin by cathepsin B, a lysosomal peptidase expressed on the surface of lymphocytes after degranulation. Sangster et al. (127) showed that cathepsin B was expressed in samples collected from patients with metastatic malignant melanoma of the head and neck. Subsequently, Yang et al. (128) reported that cytoplasmic cathepsin B expression was present in 34.6% of patients with oral squamous cell carcinoma. It was also shown that cathepsin B expression was positively correlated with lymph node metastasis and higher tumor stage, but not with tumor size and distant metastasis. Moreover, patients with positive cathepsin B expression also had shorter survival. Another mechanism involves direct granzyme inactivation due to the activity of proteins called serpins (111, 129). It has been shown that the protein responsible for granzyme B inactivation is Serpin B9, and data indicate that this protein is present in 4% of head and neck cancers (130). For example, van Kempen et al. (131) reported that expression of the granzyme B inhibitor Serpin B9, but also granzyme H – Serpin B1 and granzyme M – Serpin B4 were expressed in squamous cell carcinoma of the pharynx and larynx, while they were not expressed in healthy tissues. Interestingly, there was also no difference in expression between HPV-positive and HPV-negative tumor. The next strategy to protect tumor cells from immune cell cytotoxicity is cell cycle switching. Sun et al. (132) showed that activation of the G2/M checkpoint involving WEE1 kinase caused cell cycle arrest in oral squamous cell carcinoma cells which protected against granzyme B-induced apoptosis. In contrast, blocking WEE1 kinase with the specific inhibitor AZD1775 resulted in increased killing of cancer cells by CTL cells. It is also interesting to note that cancer cells can evade cytotoxicity via autophagy. Autophagy is known to be involved in anti-tumor immunity by promoting antigen presentation and the differentiation, maturation and survival of immune cells in the tumor microenvironment (133). However, on the other hand, this process may also contribute to promoting cancer cell survival, including through autophagic degradation of granzyme B (134).

Conventional therapies face significant challenges related to tumor resistance to treatment and immune evasion, which result in disease progression. Available forms of immunotherapy can be divided into specific (e.g., ‘immunological vaccines,’ monoclonal antibodies, CAR-T cells, etc.) and non-specific (immunostimulants, cytokines, modified NK cells, and others). The targets for the drugs used in various cancers include cytokines (e.g., aldesleukin – an interleukin-2 blocker) as well as tumor growth factors (e.g., sargramostim – a GM-CSF blocker). Gene therapy trials are also being conducted using these drugs. One of the key factors influencing the effectiveness of cancer therapy is the tumor immune microenvironment (TIME), which consists of T lymphocytes, NK cells, as well as tumor-associated macrophages (TAMs) and tumor-associated neutrophils (TANs). In the immune response to process of cancer, some lymphocytes infiltrate tumor and stay inside its tissues as tumor-infiltrating lymphocytes (TILs). Their important ability is to identify multiple immune sites of cancer cells, which can be obtained, modified and infused back into the body, to cure cancer. Moreover, in the contrast to other immunocellular therapies, like CAR-Ts and TCR-Ts, TILs do not need to be modified to recognize certain site of cancer cell. Actually, TIL therapy is used mainly to treat melanoma and few solid cancers (130, 135). It is well known that the induction of PD-L1 by inflammatory factors within the tumor microenvironment promotes immune evasion and may be one of the most significant factors affecting the therapeutic efficacy of PD-1/PD-L1 inhibitors (130). Therapies aimed at remodeling the TIME and restoring its immunostimulatory function represent a promising direction of research (136). However, it is also that treatment with PD-1/PD-L1 inhibitors demonstrates only partial efficacy (137). Another method is to kill cancer cells via transferring specific genes into patient’s T cells, which is named tumor-specific T cell receptor (TCR) therapy. In this, naturally produced TCRs are selected complementarily to cancer cells, amplified and transferred back into T cells obtained from patients. Clinically, peripheral blood T cells are often obtained by leukapheresis, modified genetically and transferred back via gam-ma-retroviral or lentiviral vectors (138, 139) or using transposon system, such as Sleeping Beauty or Crispr/Cas9 based technology (139).

#### 5.1.4 Therapeutic potential of granzyme B and its application in immuno-oncology

The clinical potential of GZMB is being explored due to its ability to induce apoptosis in cells that have developed resistance to other therapies. Therefore, the use of GZMB in cancer therapy is particularly promising in cases where traditional treatments, such as chemotherapy and radiotherapy, fail to eliminate all malignant cells. GZMB has demonstrated efficacy in a variety of cancer types, including head and neck cancers, by specifically attacking cancer cells while sparing healthy tissue (140). Zhu et al. (141) conducted a study using tongue squamous cell carcinoma cells showing that granzyme B treatment had an inhibitory effect on tumor growth by reducing the level of cell proliferation. This study also analyzed the use of trichosanthin (TSC) treatment, which at low doses increases surface expression of CI-MPR, which is a receptor for granzyme B (142). Thus, the use of combined granzyme B/TSC treatment resulted in an increase in the intensity of tumor growth inhibition compared to treatment with either drug alone. Moreover, it was reported that there was a decrease in the expression of a marker of angiogenesis (VEGF-A) in the tumor after combined treatment, confirming the involvement of the VEGF pathway in the anti-angiogenic effect of granzyme B and TCS (143).

However, one of the challenges of using GZMB in cancer therapy is its inactivation by Serpin B9 (PI-9), a natural inhibitor of GZMB that is often overexpressed in cancer cells. This overexpression allows cancer cells to avoid destruction by the immune system by neutralizing the pro-apoptotic effects of GZMB. Overcoming this inhibition is the subject of active research, which focuses on modifying GZMB to make it resistant to PI-9 or reducing PI-9 levels in cancer cells (144).

In addition to its therapeutic potential, GZMB is also being investigated as a biomarker for predicting response to immunotherapy. Elevated levels of GZMB in tumor infiltrating lymphocytes (TIL) correlate with better clinical outcomes in patients under-going therapy with immune checkpoint inhibitors. This suggests that measuring GZMB levels may help identify patients who are more likely to benefit from immunotherapy (145, 146).

Granzyme B is often indicated as a potential ideal biomarker for immune response (27), especially applications in monitoring of tumor immunotherapy. GZMB may be also a promising therapeutic tool in cancer treatment, by i.e. delivery of a cDNA fusion construct encoding an active form of GZMB into cancer cells, or by combining GZMB with other types of therapeutic agents or other treatments (19).

Granzyme B is crucial for the cytotoxic activity of NK cells, which play an important role in the innate immune response against tumor. NK cells, derived from CD34+ hematopoietic stem cells, can be classified into CD56dim and CD56bright subsets. The former subset, responsible for cytotoxic functions, releases granzyme B and perforin upon recognition of tumor cells (147). The activation of NK cells is regulated by different receptors, allowing them to detect cells lacking MHC I expression. Furthermore, HNSCC cancer cells that overexpress heat shock protein 70 (Hsp70) become more susceptible to lysis by NK, confirming the important role of granzyme B in cancer apoptosis. Recent studies on the engineering of NK-92 cell lines show their therapeutic potential in HNSCC, as they can maintain their cytotoxic capacity even under hypoxic conditions (148).

Studies by Yin et al. (149), reveal its potential for granzyme B as a diagnostic and prognostic marker. The expression of granzyme B, which is closely related to natural killer (NK) cell activity, can significantly affect the outcome of patients with these cancers. The study by Kim et al. (150) focused on angiocentric lymphoma of the head and neck, in which 57 patients were examined. The results indicated that more than 60% of them had tumor positive for granzyme B, suggesting a strong association of this expression with tumor characteristics. In addition, the co-occurrence of granzyme B with CD56 indicates a potential NK cell origin, and high granzyme B expression correlates with a higher risk of disease recurrence and a lower overall survival rate, highlighting its importance in treatment strategies and patient monitoring.

A study by Yin et al. (149) evaluated granzyme B levels in the context of the efficacy of local administration of anti-PD-L1 antibodies in the treatment of oral squamous cell carcinoma. Tumor-bearing mice received both standard doses (200 mg) and tenfold lower doses (20 mg) administered directly to the tumor. The results showed that both methods were comparable in terms of anti-tumor efficacy, with the group receiving the local low dose showing higher levels of granzyme B (p < 0.05), suggesting better activation of cytotoxic T cells. Increased granzyme B expression in response to local antibody administration may improve the immune response with a reduced risk of adverse effects. A study by Kim et al. (150) analyzed the association between granzyme B expression and clinicopathological features of patients with angiocentric head and neck lymphomas and the impact of this expression on treatment outcomes. Of the 57 patients studied, more than 60% had granzyme B-positive tumor that co-expressed CD56, suggesting an association with NK cells or activated cytotoxic T cells. Although there were no significant differences in histopathological features, the Epstein-Barr virus genome was more frequently detected in the positive group. Patients in this group had a higher risk of relapse and lower overall survival.

The collected studies clearly demonstrate the importance of granzyme B in the diagnosis and prediction of treatment outcomes of patients with head and neck cancer. High granzyme B expression may be associated with poorer treatment outcomes, making it a promising marker for therapeutic strategies. Future research should focus on further exploring the mechanisms of action of granzyme B and its role in cancer immunotherapy.

Studies on granzyme B in cancer cell apoptosis have shown that these cells can be resistant to apoptosis induced by this enzyme. This resistance may be related to the expression of the serine protease inhibitor (Serpin) PI-9 or its mouse counterpart SPI-6, which inactivate the action of granzyme B (151). Normally, PI-9 is found in immunologically privileged sites such as lymphoid tissue and on T lymphocytes and NK cells, presumably serving a protective function against immune cell self-destruction (152, 153). Clinical studies indicate that PI-9 expression in tumor cells is associated with poorer treatment outcomes in patients with metastatic melanoma (152).

*In vitro* studies, such as those by Xiu-Ying et al. (154) showed that the induction of apoptosis by granzyme B in cancer cells requires the presence of the pore-forming protein perforin. Perforin is crucial for the internalization of granzyme B into target cancer cells, allowing it to effectively induce the apoptosis cascade. Importantly, co-expression of both perforin and granzyme B was found to dramatically reduce Hep-2 cancer cell proliferation, indicating a synergistic effect of these two key cytotoxic effectors in inhibiting tumor growth.

In addition, many cancers, including head and neck cancer, contain mutations in key cell cycle regulatory proteins such as p53, p21 and Rb (155, 156). These genetic alterations may lead to an increased dependence of cancer cells on the G2/M cell cycle checkpoint, which in turn affects their response to DNA damage and their ability to effectively repair such damage (157). More recent studies suggest that the combination of granzyme B and the inflammatory cytokine TNF-α can activate this G2/M checkpoint, potentially reducing the susceptibility of these cancer cells to immune-mediated elimination (137, 158). This highlights the importance of understanding the complex interplay between cell cycle regulation and the immune response in the context of developing more effective immunotherapeutic strategies against head and neck malignancies. Interventions aimed at reversing G2/M cell cycle arrest, such as Wee1 kinase inhibition, have shown promising results in increasing the sensitivity of tumor cells to T-cell killing, both *in vitro* and *in vivo* (137, 159). These findings open new possibilities in the design of therapeutic strategies that could improve the efficacy of granzyme B-based immunotherapy. By targeting the G2/M checkpoint, these interventions may increase the susceptibility of tumor cells to the cytotoxic effects of granzyme B, potentially overcoming resistance mechanisms and enabling more efficient immune elimination of tumor cells.

#### 5.1.5 Emerging roles of granzyme B in various cancers and age-related pathologies

Recent research has focused on GZMB in the context of gastric cancer (GC), where its expression patterns and functions are being analyzed. Techniques such as quantitative real-time polymerase chain reaction, western blotting and immunohistochemistry have shown that GZMB mRNA and protein levels are significantly elevated in GC tissues, correlating with tumor progression (160). GZMB knockdown experiments suggest that its inhibition leads to a slowing of GC cell proliferation and migration, which is associated with a decrease in epithelial-mesenchymal transition (EMT) markers. On the contrary, overexpression of GZMB by plasmid transfection increases these malignant properties, highlighting its role as a promoter of growth and migration in gastric cancer, which has been confirmed in both *in vitro* and *in vivo* studies. These findings open possibilities for therapies targeting the molecular pathways of GZMB in the treatment of gastric cancer (161).

In the context of ovarian cancer (OC), which is one of the leading causes of cancer-related deaths in women, the development of effective therapies is crucial. Despite advances in maintenance treatment, a significant proportion of patients show limited response to treatment. GZMB-based protein fusions targeting key molecules in OC have shown efficacy against both sensitive and resistant cell lines. These constructs have not shown resistance to chemotherapy, suggesting their potential role in overcoming resistance mechanisms (162). GZMB also has potential as a biomarker for monitoring immune responses in cancer therapy. The imaging probes developed that respond to GZMB enable accurate assessment of immune activity, which may improve therapeutic decision-making and minimize side effects associated with excessive immune responses. These innovative approaches have shown promising results in preclinical studies (151, 163).

Recent studies have linked GZMB expression to age-related pathologies, identifying its presence in immune cell populations and its role in promoting inflammation associated with biological ageing (164). Environmental factors such as UV exposure and an unhealthy diet can induce GZMB, while its inhibition attenuates age-related disease phenotypes, suggesting its multifaceted role in both cancer and age-related conditions (165).

Innovative approaches to enhance the therapeutic efficacy of GZMB in solid tumor include systems designed to co-deliver GZMB and perforin to promote tumor cell apoptosis and inhibit tumor cell growth (166). These nanocapsules, which are responsive to the tumor microenvironment, represent a novel strategy for enhancing intracellular delivery of GZMB, potentially increasing its therapeutic effect (167). In cervical cancer (CC), the use of an immunotoxin with GZMB conjugated to an affibody targeting HPV16-infected cells showed significant growth inhibitory effects, blocking EMT and inducing apoptosis and pyroptosis. This approach demonstrates the versatility of GZMB in effective tumor control with minimal risk of systemic toxicity.

The accumulated results highlight the multifaceted role of GZMB in different types of cancer and its potential as a therapeutic target. Continued research into the mechanisms and applications of GZMB in cancer treatment holds promise for improving clinical out-comes and increasing patient survival rates (168).

### 5.2 Melittin

Melittin has garnered significant interest in cancer research due to its potent cytotoxic and anti-proliferative properties. In the field of oncology, melittin’s ability to selectively target cancer cells while sparing normal tissue has made it a promising candidate for therapeutic applications, particularly in the treatment of solid tumors, including head and neck cancers (30, 169). Melittin exerts its anti-cancer effects primarily through the disruption of cellular membranes and the induction of apoptosis. The amphipathic nature of melittin allows it to embed into lipid bilayers, forming pores that disrupt the membrane integrity of cancer cells. This disruption leads to an influx of ions, loss of cellular homeostasis, and cell lysis (96). Additionally, melittin can induce apoptosis by activating mitochondrial pathways, which involve the release of cytochrome c and the subsequent activation of caspases, essential mediators of programmed cell death (82). Interestingly, melittin has shown a higher degree of selectivity towards cancer cells compared to normal cells (169). This selectivity is attributed to the altered lipid composition of cancer cell membranes, which are often enriched in negatively charged phospholipids. These lipid changes enhance the binding affinity of melittin, making it more effective against malignant cells (170). Such specificity is crucial for the potential application of melittin in targeting head and neck cancers, which often present aggressive and treatment-resistant phenotypes. In the context of head and neck cancers, melittin has demonstrated promising results in preclinical studies (92, 120, 171). It has been shown to inhibit cell proliferation, migration, and invasion, all critical factors contributing to tumor progression. By targeting the cancer cells’ membrane integrity and inducing apoptosis, melittin effectively reduces tumor growth and metastatic potential. The role of melittin in modulating angiogenesis, the physiological process responsible for the formation of new blood vessels supplying nutrients to tumors, has also been the subject of investigation (171).

Melittin can induce cancer cell death through both direct mechanisms (including membrane destabilization and caspase activation) and indirect pathways, such as immunogenic cell death (ICD), which leads to the activation of an immune response (170). In preclinical studies, the combination of melittin with a CpG ligand and an oncolytic virus resulted in strong immune activation and effective elimination of both primary tumor and distant metastases in a murine breast cancer model, confirming the potential of this combination in cancer immunotherapy (171). However, the clinical application of melittin is limited by its non-specific toxicity, including hemolytic activity. In response to these challenges, advanced nanoparticle-based delivery systems have been developed, al-lowing for targeted administration of melittin directly to tumor cells, thereby reducing side effects and enhancing therapeutic efficacy (170). Currently, strategies are also being developed to utilize melittin as a synergistic component alongside other therapies, including immune checkpoint inhibitors. In addressing the limitations posed by its non-specific toxicity, nanocomplexes combining melittin with a photosensitizer in organic–inorganic hybrid nanocarriers have been designed (170–172). This approach enables efficient delivery of melittin directly to tumor cells while minimizing damage to healthy tissues. Furthermore, its combination with photodynamic therapy enhances treatment efficacy by inducing an immune response against the tumor. Preclinical studies have shown that this therapeutic combination leads to significant tumor growth inhibition and a reduction in metastatic risk, positioning melittin as a highly promising candidate in modern cancer immunotherapy strategies (173).

## 6 Conclusion

Granzyme B (GZMB) and melittin are molecules with unique and distinct mechanisms of action that, in the context of head and neck cancers (HNSC), may serve as potentially complementary therapeutic tools.

GZMB is a serine protease released by cytotoxic T lymphocytes and NK cells, playing a key role in the elimination of cancer cells through the induction of apoptosis. It is also involved in remodeling the tumor microenvironment and can modulate the immune response, including in the context of the immunosuppressive activity of Tregs and plasmacytoid dendritic cells (pDCs).

On the other hand, melittin—a component of bee venom—acts through destabilization of cell membranes, induction of oxidative stress, and activation of apoptotic pathways. However, its clinical application is limited by issues of toxicity and lack of selectivity.

Despite their opposing properties, the combination of GZMB’s high selectivity with melittin’s non-specific but potent cytotoxicity may lead to a synergistic therapeutic effect.

Although this hypothesis remains unconfirmed experimentally, it suggests an intriguing direction for future research. This could be particularly important in head and neck cancers, where GZMB expression has been associated both with favorable prognosis and with potential involvement in immunosuppressive mechanisms. Understanding this complexity may enable the use of GZMB as a biomarker of therapeutic efficacy.

In the future, the development of therapeutic strategies involving GZMB and melittin will require the design of effective delivery systems (e.g., nanoparticles, targeted carriers), as well as preclinical studies on their combined application.

At the same time, attention should be given to resistance mechanisms (e.g., PI-9 overexpression, cathepsin B activity, or cell cycle blockade), which may limit the effectiveness of GZMB, and to strategies for overcoming them. An integrated approach combining immunotherapy, tumor microenvironment modulation, and targeted drug delivery may enable the development of effective anticancer therapies based on these two molecules.
